# Prognosticating accelerated deterioration in skeletally mature adolescent idiopathic scoliosis curves of 40–50° using uniplanar radiographic measures of axial rotation

**DOI:** 10.1007/s43390-024-00949-1

**Published:** 2024-08-15

**Authors:** Graham Ka-Hon Shea, Samuel Yan-Lik Ng, Changmeng Zhang, Guodong Wang

**Affiliations:** 1 Department of Orthopaedics and Traumatology, The University of Hong Kong, Hong Kong, China; 2 Shandong Provincial Hospital Affiliated to Shandong First Medical University, Jinan, China

**Keywords:** Adolescent idiopathic scoliosis, Rotation, Nash–Moe, Rib Index, Prognostication

## Abstract

**Purpose:**

The management of adolescent idiopathic scoliosis (AIS) curves between 40 and 50° is controversial. Here, we investigated the prognostic significance of simple radiographic rotational parameters to identify curves of this magnitude with accelerated deterioration following skeletal maturity.

**Methods:**

Seventy-three patients were identified with AIS and Cobb angles of the major curve between 40 and 50° at skeletal maturity. We defined fast progressive curves as those increasing by ≥ 2° per year after skeletal maturity. From the apical vertebra of the major curve upon presentation and skeletal maturity, we determined the modified Nash–Moe index (×100), and from thoracic major curves, the Rib Index. *T* tests were performed to compare fast-progressive curves with those that deteriorated by < 2° per year. Receiver operator characteristic (ROC) curves were plotted to establish optimal cutoffs, sensitivity, and specificity measures for rotational parameters.

**Results:**

The average duration of follow-up post was 11.8 ± 7.3 years. Thirteen out of seventy-three patients were fast progressors. The modified Nash–Moe index was similar between groups at presentation (*p* = 0.477) but significantly higher in fast progressors than non-fast progressors at maturity for major thoracic curves (25.40 ± 6.60 vs. 19.20 ± 4.40, *p* < 0.001). Rib Index values were also higher among fast progressors at skeletal maturity (2.50 ± 0.90 vs. 1.80 ± 0.60, *p* = 0.026). An ROC curve for a modified Nash–Moe index of 0.235 for thoracic curves achieved an area under the curve (AUC) of 0.76 for discriminating fast progressors. A threshold of 1.915 for Rib Index at maturity achieved an AUC of 0.72 for discriminating fast progressors. In combining both rotational parameters, an AUC of 0.81 was achieved.

**Conclusion:**

These simple rotational parameters may be useful to predict fast progression in 40–50° AIS curves following skeletal maturity indicated for early fusion, but further validation upon larger cohorts and non-thoracic major curves is required.

## Introduction

Adolescent idiopathic scoliosis (AIS) is a spinal deformity that manifests during puberty, with a population prevalence ranging from 2 to 4% [1]. Long-term observational studies have indicated a preponderance of curves exceeding 50° to deteriorate [2], and therefore surgical intervention is indicated. However, the management of curves measuring between 40 and 50° at skeletal maturity remains controversial. Some spinal surgeons suggest observation, while others advocate for pre-emptive fusion due to anticipated risk of curve progression.

It has recently been demonstrated upon longitudinal observation of the decade following skeletal maturity that over 60% of curves in the 40–50° range continue to progress [3]. Most curves progress by 1–2° per year, and the proportion of fast progressors (> 2^o^ progression per year) consisted of less than 20% of the study cohort. Nevertheless, it is essential that this subgroup of subjects with fast progression are identified early, as they would benefit the most from early surgical fixation. While coronal imbalance and vertebral wedging were identified as prognostic indicators for curve deterioration, the role of vertebral rotation remains unknown for this 40–50° cohort.

Here, we investigate the relationship between axial rotation of the major curve and post-maturity progression rate in subjects with AIS and major curves between 40 and 50° at the time of skeletal maturity. We utilized a modified Nash–Moe index [4] which was quantifiable as a continuous variable, as well as Rib Index [5, 6], as simple radiographic indices approximating vertebral rotation. The findings of this study contribute to the development of personalized treatment strategies for AIS patients with curves within the tenuous 40–50° range.

## Methods

### Study design and population

This retrospective study was conducted at a tertiary referral center. Patients attending the scoliosis clinic between 2015 and 2020 were recruited based on (1) a diagnosis of AIS, (2) major curve Cobb angle of 40–50° upon standing posteroanterior (PA) radiographs taken at skeletal maturity, (3) no surgical intervention prior to skeletal maturity, and (4) sufficient follow-up to ascertain curve progression (see below). We excluded patients who were over 20 years of age at first presentation. Skeletal maturity was defined as a distal radius and ulna (DRU) classification of R10 U9 and above in cases with a hand X-ray. In cases without a hand X-ray, skeletal maturity was defined by a Risser score of 4, 24 months post-menarche in females and/or an absence of height increase upon follow-up [3].

### Determination of post-maturity curve trajectory

Clinical practice at our tertiary referral center is to offer yearly follow-up to skeletally mature patients with AIS and a major curve in the 40–50° range due to the risk of subsequent curve progression. These patients received yearly monitoring via standard standing whole spine radiographs prior to 2016, and via sterEOS™ biplanar X-rays from 2016 onwards, taken during the morning scoliosis clinic. Curve progression was defined as ≥ 5° increase in Cobb angle following skeletal maturity. Among those with progression, we defined “fast progression” as those with a Cobb angle progression rate of > 2° per year. The rate of Cobb angle progression was calculated by dividing the absolute change in Cobb angle from the first follow-up post-maturity to the latest follow-up, by the time interval between these two follow-up visits. For patients receiving spinal fusion, Cobb angle magnitudes were assessed on preoperative radiographs obtained closest to the operation date.

### Measurements of rotational indices

#### Evaluation of vertebral rotation by the modified Nash–Moe method

**Fig. 1:**
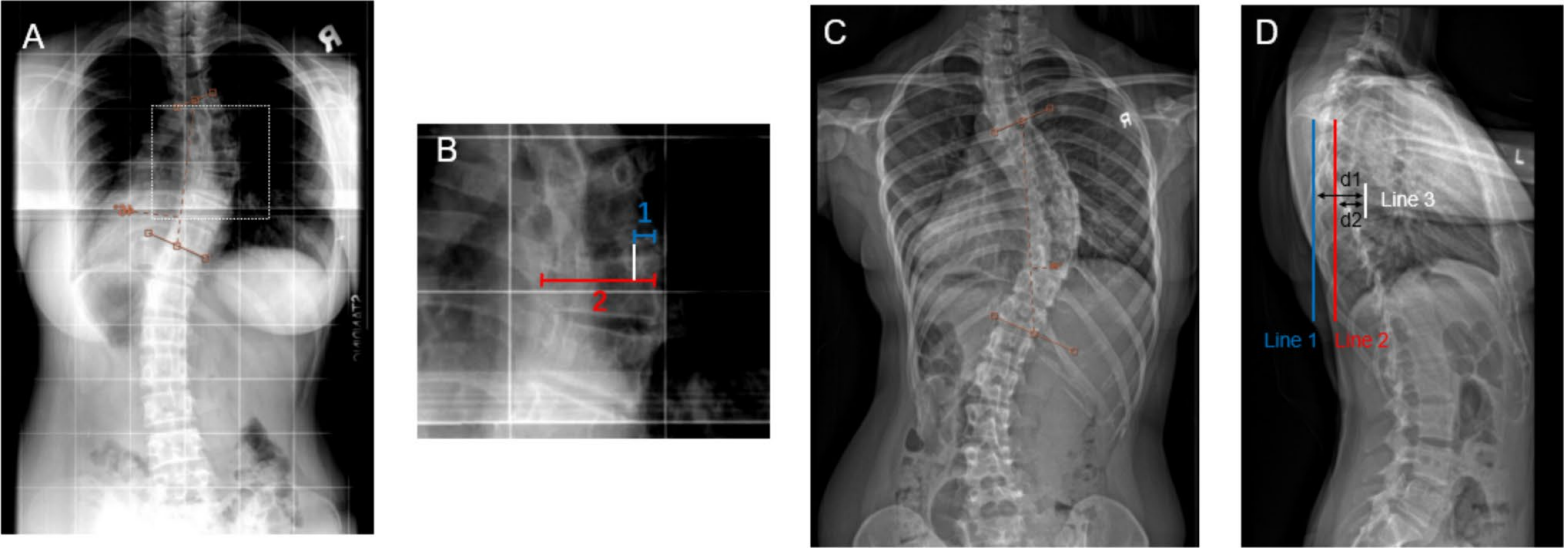
Measurement of axial rotation via modified Nash–Moe method and Rib Index. **A** A right thoracic curve of 46° spanning T5–T10, with the major curve apex enlarged and shown in (**B**). Upon (**B**), the axial rotation of the apical vertebra was estimated by dividing the distance from the lateral vertebral border to the central aspect of the convex pedicle shadow (1) by the width of the vertebra over the mid-vertebra (2). **C**, **D** Posteroanterior and lateral standing X-rays of a right thoracic curve of 46° spanning T5–T12. **D** Line 1 corresponds to a vertical tangential line to the most posterior rib point, while Line 2 corresponds to a vertical tangential line to the least projected rib point at the same height. Line 3 is a vertical line drawn against the posterior vertebral margin at a height corresponding to prior rib point measurements. The distance between Line 3 and Line 1 (d1) is divided by the distance between Line 2 and Line 3 (d2) to obtain the Rib Index

X-rays of the spine were retrieved from the picture archiving and communication system (PACS). The Nash–Moe method [4] originally classified vertebral rotation into five sectors, based on the location of the pedicle in relation to the vertebral body width. Upon modification, the position of the convex pedicle may be quantified as a continuous variable in relation to the position of its center from the vertebral edge over the mid-vertebra (a), which is divided by the vertebral width (b) [7] and multiplied by 100. The method of measurement is illustrated in Fig. 1A. Only the apical vertebra of the major curve (curve with largest Cobb angle) was considered.

#### Evaluation of the Rib Index

The double rib contour (DRC), which we quantify in major thoracic curves via calculation of Rib Index, has been described as a means to measure rib hump size upon lateral standing radiographs [5, 6]. In brief, the most extended (posterior) rib point was identified upon lateral films of the thoracic spine and a tangential line drawn vertically to intersect this point (Line 1). Next, the least projected rib point was identified at a location perpendicular (at the same height) to that of the first line, with another vertical line drawn to intersect this point (Line 2). The corresponding vertebra at the height of the most extended and least projected rib point is identified, and a vertical line drawn against its posterior vertebral margin (Line 3). The distance between Line 3 and Line 1 (d1) is divided by the distance between Line 2 and Line 3 (d2). The Rib Index (d1/d2) represents a measurement of rib hump size. The method of measurement is illustrated in Fig. 1B.

### Statistical methods

For comparison between two groups of continuous variables with a normal distribution, we utilized the unpaired *T* test. For comparisons of categorical variables, the Pearson Chi-square test was utilized or alternatively, Fisher’s exact test when expected cell frequencies were < 5. The intra-class correlation coefficient was calculated as a measure of intra-observer and inter-observer reliability between two measurements and two observers, respectively. For all statistical tests, a one-tailed *p* value of < 0.05 was considered statistically significant. Receiver operating characteristic (ROC) curves were plotted to determine the area under the curve (AUC) for radiological parameters with significant differences between groups. Cutoff values that optimized sensitivity and specificity toward the prognostication of fast progressive curves were determined with the Youden J statistic. Missing data were excluded from analysis.

## Results

### Patient demographics

A total of 73 patients fulfilled the recruitment criteria, among which 60 were non-fast progressors, and 13 were fast progressors. There was a female preponderance among the entire cohort (61 females, 12 males) which did not differ in non-fast and fast progressor subgroups (*p* = 0.124). The average follow-up duration until latest follow-up or surgery was 13.8 ± 7.3 in non-fast progressors as compared to 4.9 ± 2.8 among fast progressors which was statistically significant (*p* < 0.001). All 13 patients among fast progressors had received surgery by latest follow-up, as compared to 15/60 among non-fast progressors (*p* < 0.001), and this disproportion in surgical intervention accounted for the difference in follow-up period. Thoracic major curves were more prevalent than thoracolumbar or lumbar major curves although this did not significantly differ between subgroups (*p* = 0.858). Among non-fast progressors and fast progressors, age at presentation (14.0 ± 3.0 vs. 13.8 ± 1.6, *p* = 0.420), Cobb angle at presentation (33.9° ± 8.4° vs. 34.4° ± 8.6°, *p* = 0.432), age at skeletal maturity (17.0 ± 2.3 vs. 16.5 ± 1.6, *p* = 0.220), and Cobb angle at skeletal maturity (44.6° ± 3.5° vs. 45.4° ± 3.3°, *p* = 0.224) did not differ. These results are summarized in Table 1.

**Table 1:** Demographics and rotational parameters of fast and non-fast progressors

	Non-fast progressors	Fast progressors	*p* value
N-number	60	13	NA
Gender (F/M)	52/8	9/4	0.124
Follow-up (years)^#^	13.8 ± 7.3	4.9 ± 2.8	< 0.001
Initial presentation			
Age	14.0 ± 3.0	13.8 ± 1.6	0.420
Cobb angle	33.9° ± 8.4°	34.4° ± 8.6°	0.432
Skeletal maturity			
Age	17.0 ± 2.3	16.5 ± 1.6	0.220
Cobb angle	44.6° ± 3.5°	45.4° ± 3.3°	0.224
Major curve (thoracic/other)	40/20	9/4	0.858
Modified Nash–Moe			
Presentation	17.3 ± 5.5	17.2 ± 6.5	0.477
Maturity (all curves)	21.6 ± 6.8	26.6 ± 6.9	< 0.01
Maturity (thoracic curves)	19.2 ± 4.4	25.4 ± 6.6	< 0.001
Rib Index			
Presentation	1.7 ± 0.3	N.A	N.A
Maturity (thoracic curves)	1.8 ± 0.6	2.5 ± 0.9	0.026

### Rotational indices

The modified Nash–Moe index was similar upon presentation in non-fast progressors and fast progressors (17.3 ± 5.5 vs 17.2 ± 6.5, *p* = 0.477). Upon X-rays taken at skeletal maturity, however, the Nash-Moe index was significantly higher in rapid progressors than non-rapid progressors (26.6 ± 6.9 vs. 21.6 ± 6.8, < 0.01). These differences remained statistically significant and were more pronounced upon comparison of thoracic curves only (25.4 ± 6.6 vs. 19.2 ± 4.4, *p* < 0.001). However, upon considering non-thoracic major curves, differences in modified Nash–Moe index were insignificant (22.6 ± 8.3 vs. 20.8 ± 6.1, *p* = 0.334).

**Fig. 2:**
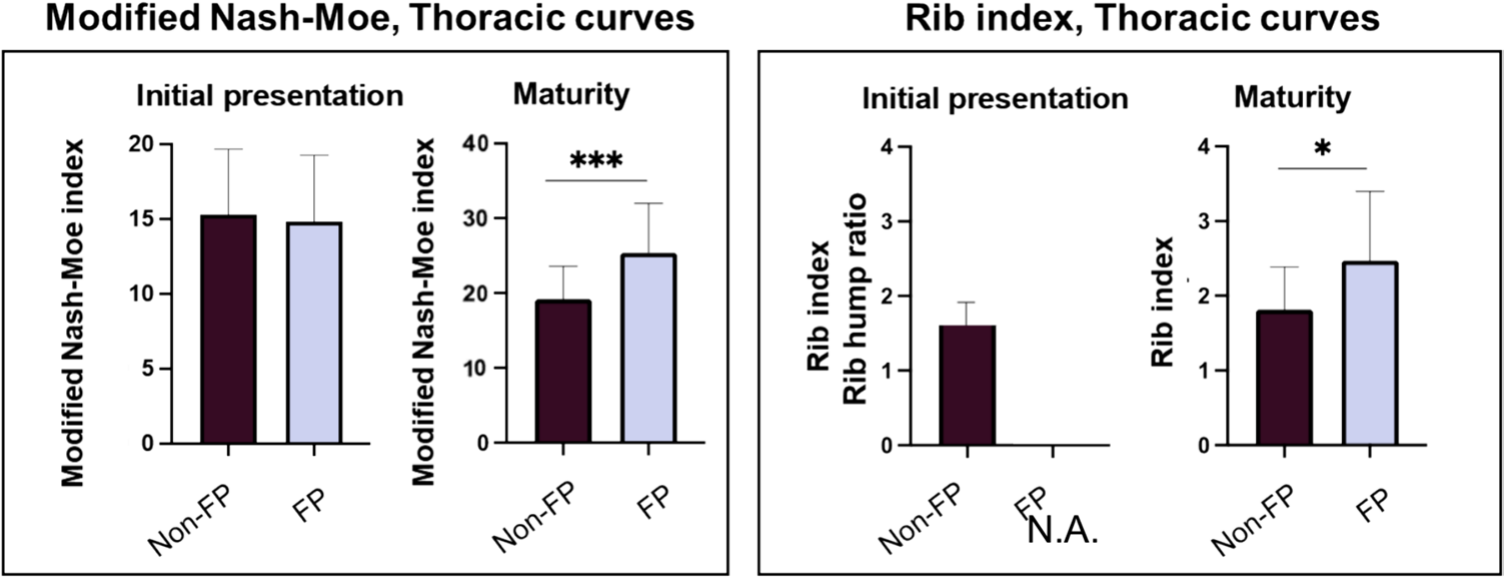
Bar charts of rotational parameters during presentation and maturity. Modified Nash–Moe and Rib Index measurements at presentation and skeletal maturity. * *p* < 0.05, *** *p* < 0.001

With regards to Rib Index, the number of lateral X-rays were insufficient to facilitate comparison at presentation, since many cases with an ascertained natural history presented early at our center when only PA films were required for routine radiological monitoring. Upon assessment of thoracic curves at maturity, however, Rib Index was significantly increased in fast progressors (2.5 ± 0.9 vs. 1.8 ± 0.6, *p* = 0.026). These findings are summarized in Table 1 and Fig. 2.

The intra-class correlation coefficient for Nash–Moe grading was 0.975 (97% CI: 0.963–0.983) and that of the Rib Index was 0.995 (95% CI: 0.992–0.997). The inter-class correlation coefficient for Nash–Moe grading was 0.876 (95% CI: 0.817–0.916) and that of the Rib Index was 0.950 (95% CI: 0.921–0.969). Thus, both intra- and inter-class correlation achieved very good reliability.

### Receiver operator characteristic (ROC) curves in relation to rotational indices

**Fig. 3:**
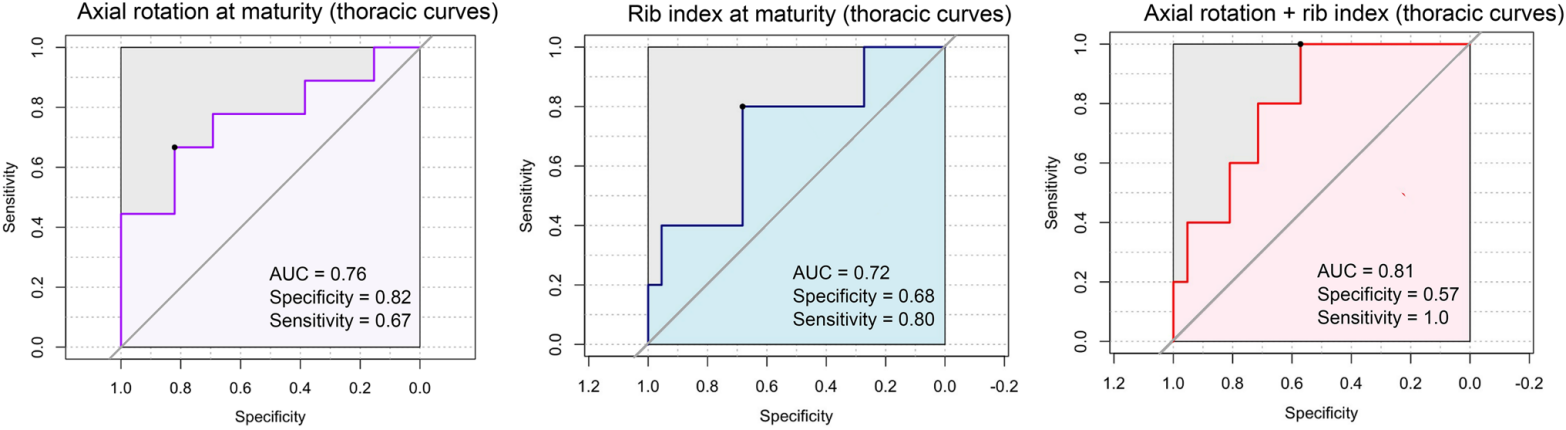
ROC curves for rotation parameters and fast progression. Cutoff values that optimized sensitivity and specificity toward the prognostication of fast progressive curves were determined with the Youden J statistic

When considering the modified Nash–Moe index for thoracic curves, a threshold of 23.6 achieved an AUC of 0.76 with specificity of 0.82 and sensitivity of 0.67 for identifying fast progressors. When considering Rib Index at maturity for thoracic curves, a threshold of 1.9 achieved an AUC of 0.72, specificity of 0.68, and sensitivity of 0.80. Combining the modified Nash–Moe index (threshold of 13.9) together with Rib Index (threshold of 2.1) resulted in an AUC of 0.81, with a sensitivity of 1.0 and a specificity of 0.57 in identifying fast progressors. These results are shown in Fig. 3.

## Discussion

The management of AIS curves of 40–50° magnitude at skeletal maturity has remained controversial. Data on natural history have recently emerged to demonstrate that although over 60% of such curves progress, most do so slowly at between 1 and 2° per year [3], and therefore may be monitored yearly with intervention offered in response to documented radiological deterioration. The objective of this study was to investigate further radiological determinants of fast progressors, since this group would benefit from more frequent monitoring and/or pre-emptive spinal fusion. We identified increased modified Nash–Moe values and Rib Index as simple radiological indices of rotation for thoracic curves indicating a risk for fast progression in the years following skeletal maturity.

While diagnosis is dependent on Cobb angulation upon the coronal plane, scoliosis is well recognized as a complex three-dimensional deformity. Truncal rotation is a feature of structural curves, forming the basis for the use of a scoliometer for school-aged population screening [8]. Nash–Moe described one of the earlier methods of estimating vertebral rotation based on two-dimensional radiographs that has been validated by others to demonstrate good intra-observer and inter-observer reliability [9]. More sophisticated means for two-dimensional measurements of rotational have been described, for example the Perdriolle method, whereupon a torsionmeter translates the pedicle position to a corresponding angulation of axial vertebral rotation [10]. Cerny more recently described a complicated means of determining three-dimensional orientation upon anteroposterior views obtained from traditional X-rays which exceeded the performance of the Perdriolle method in measuring rotation and approximated that of CT and MRI verification [11]. In the recent decade, measurement of axial rotation has favored the use of biplanar X-rays followed by semi-automated software reconstruction [12, 13]. Nevertheless, when considering clinical application, estimates of vertebral rotation from uniplanar films remain advantageous as measurements are quickly and easily ascertained without specialized equipment, software, and expertise.

The prognostic significance of axial rotation for AIS curve progression overall, and not upon this 40–50^o^ range alone, has been extensively characterized from prior literature. Three-dimensional apical rotation, and additionally, plane of maximal curvature and torsion, are all related to major curve rotational deformity, and have prognostic relevance at curves upon initial presentation [12, 13]. From a biomechanical standpoint, a deformity in the coronal plane has been posited to result in asymmetric loading of the vertebrae and endplate, resulting in differential growth and wedging [14]. In addition, idiopathic scoliosis has been described as a rotatory decompensation of the spine [15]. Coronal deformity is coupled with rotation, which forms the basis for derotation techniques during surgical correction [16]. Axial rotation that is disproportional or exaggerated with relation to the sagittal plane deformity may be an early indication of mechanical decompensation of spinal alignment [17]. While more literature in the past has utilized pedicle rotation for prognostication purposes, our results indicated a role for Rib Index measurements as well. The prognostic role of the Rib Index as an alternative to three-dimensional rotational measures merits further investigation in a follow-up study not limited to curves of 40–50° upon reaching skeletal maturity. Intra-observer and inter-observer measurement reliability of the Rib Index appeared to exceed that of the modified Nash–Moe index; however, rib hump measurements may be harder to accurately ascertain in curvatures < 40°. While results appear to show that increased rotation is a potentially destabilizing factor, three-dimensional reconstruction should be utilized to investigate how these simple radiological measurements correlate with axial rotation, and thereafter, progression risk.

A limitation in our study is the relative imbalance and dearth of fast progressors among the entire study cohort (13 out of 73). This limited further subgroup analysis since most major curves were thoracic in location, and the relevance of our findings needs to be delineated upon non-thoracic major curves. Our findings should be replicated upon larger sample sizes as differences in rotation between fast and non fast progressors need to be considered in light of possible discrepancies noted from intra- and inter-observer reliability measures, and across different surgical centers to demonstrate reproducibility. As data from the natural history of these 40–50° curves extended for approximately 10 years after skeletal maturity, it would be of interest to review how curves fared upon an extended period of follow-up.

## Conclusion

We identified vertebral rotation and Rib Index as simple radiological indices of vertebral rotation indicating a risk for fast progression in the years following skeletal maturity. Upon further external validation, patients with at-risk parameters may be considered to receive early surgical fixation instead of observation alone.

## Data Availability

Derived data supporting the findings of this study are available from the first author (GKHS) on request.
